# Evaluation of real-world early response of DMO to aflibercept therapy to inform future clinical trial design of novel investigational agents

**DOI:** 10.1038/s41598-020-73571-6

**Published:** 2020-10-05

**Authors:** Sandra Halim, Sarega Gurudas, Shruti Chandra, John Greenwood, Sobha Sivaprasad

**Affiliations:** 1 UCL Institute of Ophthalmology, London, UK; 2 NIHR Biomedical Research Centre, Moorfields Eye Hospital NHS Foundation Trust, University College London, 11-43 Bath Street, London, EC1V 9EL UK

**Keywords:** Anatomy, Biomarkers, Diseases, Health care, Medical research, Risk factors, Signs and symptoms

## Abstract

New clinical trials for diabetic macular oedema (DMO) are being designed to prove superiority over aflibercept when this agent is already very effective in improving visual acuity (VA) and DMO. The aim of this study was to determine the optimal inclusion–exclusion criteria for trials to aim for superiority in visual outcomes with newer agents. As Phase 1 studies are short duration, we aimed to evaluate the early response of aflibercept in a real-world cohort initiated on monthly aflibercept for 3 consecutive injections and observed the effects at 4 months. The sub-optimal responders were pre-defined based on different cut-offs for VA and central sub-field thickness (CST). 200 patients with treatment naïve DMO treated with 3 loading doses of aflibercept were included in the study. We found that those presenting with baseline VA of 35–54 ETDRS letters (n = 43) had higher proportion of sub-optimal responders compared to other categories (p < 0.001). Patients with baseline CST of less than 400 µm (n = 96) responded less well functionally and anatomically to loading dose than eyes with baseline CST of 400 µm or more (n = 104, p = 0.02), indicating that eyes with CST ≥ 400 µm is another inclusion criteria. There was minimal correlation between change in CST and change in VA at 4 months (r = − 0.27), suggesting that both these inclusion criteria are non-exclusive. However, for maximal efficacy, patients that meet both these inclusion criteria are more likely to show benefit from an alternative intervention. New trials should aim to include patients with treatment naïve DMO with VA between 35–54 letters and CST of 400 µm or more when aflibercept is used as the comparator.

## Introduction

Centre-involving diabetic macular oedema (DMO) is a sight threatening complication of diabetes characterised by macula fluid that can be qualitatively and quantitatively assessed using optical coherence tomography (OCT)^1,2^. The standard treatment of eyes with visual impairment due to DMO is repeated intravitreal anti-vascular endothelial growth factor (anti-VEGF) injections until disease stability is achieved^3–8^. In clinical trials, treatment with any of the three currently available antiVEGF agents, bevacizumab, ranibizumab or aflibercept in eyes with visual impairment due to DMO resulted in approximately 50% of eyes gaining more than 2 lines of vision over 2 years and 30% gaining 3 or more lines of vision^4,6,9^. Protocol T showed that aflibercept is more efficacious when considering the change in visual acuity (VA) over time especially in the loading phase and it is also more effective in causing resolution of the macular fluid^4^. Therefore, in clinical trials on new agents, aflibercept should ideally be used as the comparator.

Currently, several novel agents are being investigated for their efficacy in DMO. These drugs should ideally show superior response and/or durability whilst maintaining easy delivery and similar or lower costs compared to aflibercept to be more advantageous. A key challenge is the design of clinical trials for these agents. As proof-of-concept studies are short (usually 6 months duration), novel agents should ideally show an early superior VA outcome within 4–6 months for the drug manufacturers to plan a future superiority trial. Early VA responses at 12 weeks have been explored to assess long-term outcomes with conflicting results in trials of new agents aiming for superior visual outcomes^9,10^.

It is also clear from the trial design of Protocol U that there is always room for improvement in eyes with DMO when sub-optimally treated with anti-VEGF agents^10^. In Protocol U, when treated with ranibizumab loading dose before randomisation approximately 45% of the patients defined as having persistent oedema improved, suggesting that most eyes assumed to have persistent oedema respond to anti-VEGF agent when optimally treated.

There are other challenges that also need to be considered. For example, the recent analysis by Bressler et al., showed that there is no correlation between VA outcomes and changes in central subfield thickness (CST) at 12, 52, and 104 weeks after antiVEGF agents for DMO^11^. Moreover, the DRCR protocol W showed that the majority of eyes with good VA defined as Snellen 20/25 did not show a deterioration of VA over 2 years, with only about 17% needing initiation of aflibercept injections during this period^12^.

Therefore, choosing the ideal cohort to evaluate novel agents is clearly a necessity to be certain that the effects of these agents are superior to current therapies. Sub-optimal response to aflibercept in the loading phase in real-life is not well studied. Although post-hoc analysis of Phase 3 RCTs are available, these do not represent all patients with DMO especially those with good presenting VA.

The aim of this study was to determine the early response to aflibercept loading phase of 3 injections in treatment naïve DMO in real-life to extrapolate the inclusion criteria for investigating novel agents. Real-life studies are better for this study because we require an unselected DMO cohort to define the inclusion criteria and where suboptimal responses may be identified better than in the best care scenario seen in the strict protocols of a randomised controlled trial.

The research questions included:

1. What is the ideal visual acuity inclusion criterion for clinical trials on new investigational products for DMO when the comparator is aflibercept?
2. What is the central macular thickness that will potentially respond better to another treatment?
3. Are there any anatomical features that need to be excluded to ensure optimal response?

## Methods

### Study design

This was a retrospective, single-centre study. The study was approved by the Clinical Effectiveness Department, Moorfields Eye Hospital, London (Study Number 577) and informed consent from study participants was waived. The study adhered to the Declaration of Helsinki.

### Study participants

We conducted a search of the Moorfields Eye Hospital electronic patient records (Open Eyes) for patients diagnosed with new onset DMO commenced on aflibercept therapy between December 2016 and September 2019.

Centre-involving DMO was diagnosed clinically, confirmed by fundus fluorescein angiography (FFA) and spectral domain optical coherence tomography (SD-OCT). To be included in this study, the eyes had to complete monthly intravitreal aflibercept therapy for 3 months and follow-up visit at 4 months. The presence of DMO was defined as central subfield thickness (CST) ≥ 300 µm on SD-OCT (Topcon 3D-1000, Topcon, Japan) due to intraretinal oedema and/ or subretinal fluid due to DMO. Vitrectomised eyes and eyes previously treated with panretinal photocoagulation (PRP) were included. Although macular laser was not an exclusion criterion, there were no patients with previous macular laser.

Exclusion criteria for the study eye included the following: (1) any intravitreal injections of anti-VEGF agents or corticosteroids prior to initiation of aflibercept; (2) presence of other ocular disease that may confound the results (including macular degeneration, macular hole, and uveitis); and (3) patients who had inadequate retinal imaging at baseline and 4 months.

### Data collection

For eligible patients, the following baseline data were collected from their medical records: age, gender, ethnicity, type of diabetes, VA measured in Early Treatment Diabetic Retinopathy (ETDRS) letter score in clinics, SD-OCT central subfield thickness and total macular volume and severity grade of diabetic retinopathy. The VA measurements were also collected 1 month after 3 loading injections. All SD-OCT scans at baseline and after 3 loading injections were double graded for the following morphological parameters: predominant type of DMO (diffuse retinal thickening, cystoid, neurosensory detachment (NSD); cyst location (foveal if within the central 1 mm); cyst size (small if less than a quarter of retinal thickness, large if more than half of retinal thickness); disorganisation of retinal inner layers within the central 1 mm, state of subfoveal external limiting membrane (ELM) defined as absent, disrupted or intact in central 500 μm, state of subfoveal ellipsoid zone (EZ) defined as absent, disrupted or intact in central 500 μm, presence/absence of posterior vitreous detachment, presence/absence of vitreomacular abnormalities, presence/absence of epiretinal membrane (ERM), and presence of hyperreflective foci (HRF counted as present if > 20 lesions in the central 3 mm).

Limited early response was defined utilizing both functional and anatomical parameters. Limited functional response was defined as a gain of less than 5 letters and anatomical limited response was defined as < 10% decrease or increase in central subfield thickness after 3 loading injections.

### Statistical analyses

Descriptive data were presented as means and standard deviation (SD). Wilcoxon Rank test was used to compare outcome variables between baseline and follow-up visits. Baseline VA was stratified into four subgroups: 70 ETDRS letters or better; 55–69 letters, 35–54 letters and < 35 letters, and baseline and 1-month post loading VA within each subgroup were compared using Wilcoxon Rank test. All parameters were compared between baseline and one-month post loading phase. The morphological parameters were compared between those eyes that responded versus limited responders using Pearson chi-squared test or Fisher’s exact test when the expected value in any cell is 5 or less. Statistical analysis was performed using STATA software (V.15.1, STATA, Chicago, IL, USA).

## Results

Two hundred eyes that received intravitreal aflibercept loading doses for new onset DMO between December 2016 and September 2019 and had complete dataset were included in the analyses. At baseline, the mean age of the study cohort was 62.8 (SD 12.2) years, 64% were males and of multiethnic descent (40% Caucasian, 23% other Asian, 19% Indian, 11% black African and 7% others) and 91% of the cohort had type 2 diabetes.

### Analysis of visual acuity outcomes

The mean baseline VA (SD) was 62.3 (SD 15.2) ETDRS letters. The mean change in VA for the whole cohort was + 4.8 (SD 10.2) ETDRS letters after the 3 loading injections. 107 (53.5%) did not gain 5 or more letters after the loading dose. Table 1 shows the absolute VA response for the whole cohort.

**Table 1:** Responders per baseline visual acuity category.

	Baseline	After 3 injections	P-value^a^
Whole cohort (N = 200)	62.3 (15.2)	67.0 (14.7)	< 0.001
**VA subgroups**			
VA in category with baseline VA ≥ 78 letters (N = 29)	81.5 (3.2)	80.9 (5.9)	0.43
VA in category with baseline VA 70–77 letters (N = 51)	73.3 (2.4)	73.4 (7.8)	0.61
VA in category with baseline VA 55–69 (N = 68)	61.4 (4.1)	69.0 (9.7)	< 0.001
VA in category with baseline VA 35–54 letters (N = 43)	45.5 (6.0)	53.1 (12.8)	< 0.001
VA in category with baseline VA < 35 letters (N = 9)	24.3 (8.6)	38.4 (16.5)	0.03

Mean (SD) for absolute VA response in different subgroups at baseline and after 3 injections.

^a^P-values generated from Wilcoxon signed rank test.

The majority of the patients who presented with VA categories 55–69 letters (Snellen 20/80 to < 20/40) and 35–54 letters (Snellen 20/200 to < 20/80) showed significant improvement in VA. Higher standard deviation was observed in the lower VA categories. Although, the maximum VA gain was observed in the category of < 35 letters, the numbers of patients who presented in this category is low.

Table 2 shows the response in terms of change in absolute VA categories and the VA change in the limited responders. In the category presenting with 70 or more ETDRS letters, 13/80 (16.3%) dropped to a lower category. As ceiling effect is a challenge in this group, this drop of VA will apply to all treatment options and is not necessarily a drug non-response. Nearly half the patients in the 35–54 letter category remained in the same category suggesting wider variability in response in this group. Therefore, this group most likely contains the highest proportion of patients that may benefit from another agent in terms of VA improvement and less variability of VA outcomes within the cohort. Although the < 35 letter group showed a mean change in VA of 14 letters, the absolute final VA remained poor. This group also usually contains patients with structural damage to fovea or have co-existent other pathologies, explaining why the mean absolute baseline VA and outcomes are poor. From these observations, the group 35–54 letters are most appropriate to be treated with another agent.

**Table 2:** Shows the final VA in terms of absolute ETDRS letters, change in VA and central macular thickness and the characteristics of the limited responders.

Final visit	Baseline VA				P-value
	70 or more letters N = 80	55–69 letters N = 68	35–54 letters N = 43	< 35 letters N = 9	
**Final VA**					
70 or more letters (N = 112)	67/80 (83.8%)	39/68 (57.4%)	6/43 (14%)	0/9 (0%)	
55–69 letters (N = 51)	12 (15%)	24 (35.3%)	13 (30.2%)	2 (22.2%)	
35–54 letters (N = 29)	1 (1.3%)	5 (7.4%)	21 (48.8%)	2 (22.2%)	
< 35 letters (N = 8)	0 (0%)	0 (0%)	3 (7%)	5 (55.6%)	
Mean (SD) change in VA	− 0.2 (6.7)	7.5 (9.3)	7.6 (10.6)	14.1 (19.8)	< 0.001^1^
Mean (SD) change in CST	− 87.9 (95.0)	− 114.2 (96.7)	− 119.9 (145.1)	− 141.9 (170.5)	0.02^1^
Mean (SD) change in CST as a percentage of baseline CST	− 20.5 (20.0)	− 26.1 (18.4)	− 22.2 (25.1)	− 22.8 (23.6)	0.01^1^
CST < 300 μm	48 (60%)	35 (51.5%)	16 (37.2%)	2 (22.2%)	0.03^2^
CST reduction < 10%	22 (27.5%)	10 (14.7%)	15 (34.9%)	3 (33.3%)	0.08^2^
VA gain of less than 5 letters	61 (76.3%)	23 (33.8%)	19 (44.2%)	4 (44.4%)	< 0.001^2^
CST < 10% + VA gain of less than 5 letters	20 (25%)	5 (7.4%)	8 (18.6%)	2 (22.2%)	0.043^2^

*P-values generated from ANOVA (^1^) and Pearson Chi-squared test (^2^).

### Analysis of central macular thickness

**Figure 1:**
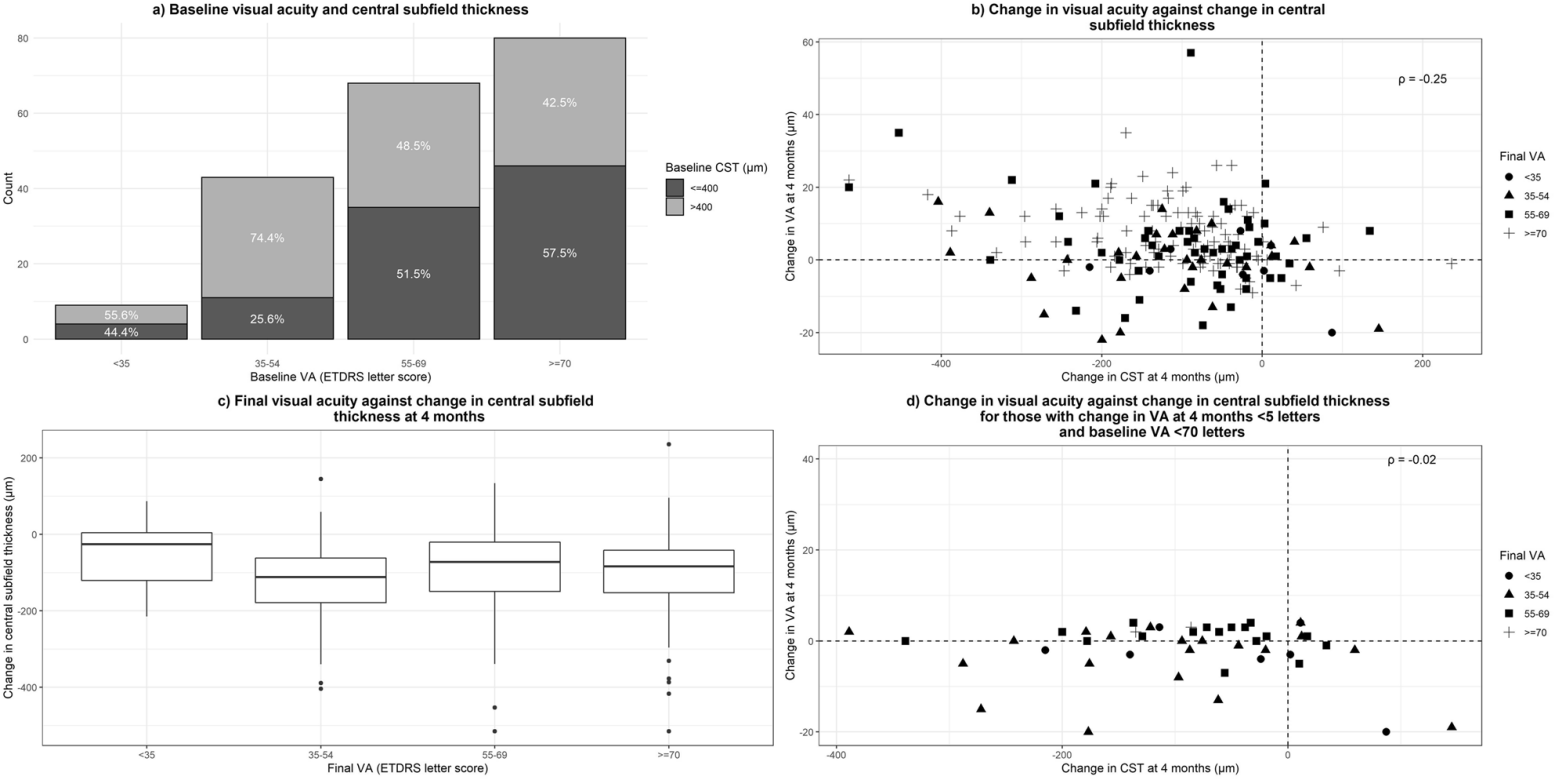
Graphs to show correlation between VA and CST. (**a**) Boxplot showing change in CST per final VA categories, (**b**) Scatterplot showing change in VA against change in CST for the whole group, (**c**) Boxplot showing change in CST per final VA category, (**d**) Scatterplot showing change in VA against change in CST for those with limited functional response and presenting VA of less than 70 ETDRS letters and (**d**) VA less than 5 letters and CST reduction of 10% or less.

Baseline CST categorised into < 400 μm and ≥ 400 μm is shown in Fig. 1. After the loading phase, 99 (49.50%) of the whole cohort had CST ≥ 300 μm and CST reduction of 10% or more was not achieved in 50 (25%) of the cohort. Patients with baseline CST < 400 μm thickness (n = 96) showed a mean gain of 3.0 (SD = 8.4) letters at final visit compared to 6.4 (SD = 11.5) letter gain in the ≥ 400 μm group (n = 104) (p = 0.02). This is because most patients with CST < 400 µm were in the better VA categories and so potential for VA improvement is limited. Significantly higher number of patients in the VA 70 letters or above and the 55–69 letter categories had a CST of < 300 μm after the loading phase, although the change in CST was modest in these categories compared to the lower categories. In contrast, 74.4% of the group with VA 35–54 letters had CST ≥ 400 µm but only 31.3% of them achieved a CST < 300 µm. This provides us with more evidence that higher VA categories, ≥ 70 letters or 55–69 letters, also have higher rates of macular fluid resolution with aflibercept and is therefore not an ideal group to evaluate a superiority in VA with another agent.

### Analysis of limited responders

When considering the whole cohort, 107 (53.5%) did not gain 5 or more letters after the loading dose. These patients had mean baseline VA of 66 letters (SD = 15.3), approximately 5 letters higher than the mean of the whole cohort and 76.3% of the patients presented with 70 letters or better. On excluding the patients with 70 or more letters at baseline, 46 patients gained less than 5 letters. In this group, 19/46 (41%) were in the 35–54 (41%) and 55–69 letter (50%) groups at baseline. However, although the 55–69 letter group had considerable numbers of non-responders based on this definition, 57.4% of patients in this group improved to 70 letters and the standard deviation was lesser than the 35–54 letter category. So, the chance of superiority in this group is lesser than the 35–54 letter group.

The mean baseline CST of the limited responders (decrease of less than 10% of baseline CST) was 382 µm (SD = 108) and not dissimilar to the mean baseline CST of 420.9 µm (SD = 115.3) for the whole group. The mean CST after 3 injections was 314.7 µm (SD = 100.7) for the whole cohort (p < 0.001), with a mean reduction of CST by 106.2 µm (SD = 112.2). In contrast, the mean CST after 3 injections in the anatomical limited responder was 391 µm (SD = 122) (p = 0.123) and the mean change of CST was only 9 µm (SD = 54.3).

### Correlation of VA and CST

In order to study the limited responders in VA and CST, we compared the overall correlation of change in CST and VA for whole cohort versus the limited responders based on VA. As previously reported, there was only a modest correlation between change in CST and change in VA at 4 months for the whole group (r = -0.25) (Fig. 1)^13^. The correlation in the limited responders with baseline < 70 letters (n = 46) was − 0.02 (Fig. 1).

### Characteristics of limited non-responders

We also compared the disease characteristics of the responders and limited non-responders defined as gaining less than 5 letters in both the whole cohort and in the 35–54 and 55–69 letters category (Table 3). There was a total of 111 patients who had baseline VA categories of 35–54 and 55–69. Except for more proliferative diabetic retinopathy patients in the non-responder group in the whole cohort, there were no obvious exclusion criteria identified. Similar analysis was done for limited response based on < 10% CST reduction and the only significant parameters that defined limited responders were presence of foveal cysts (p = 0.03) and non-intact ellipsoid zone (p = 0.04). In those with both CST < 10% and VA less than 5-letter gain, none of these parameters were statistically significant.

**Table 3:** Limited responders based on gain in visual acuity of less than 5 letters gain.

Clinical characteristics	All patients	Whole cohort		35–54 and 55–69 letter categories	
		Responders (n = 93)	Limited responders (gain of less than 5 letters) (n = 107)	Responders (n = 69)	Limited responders (n = 42)
**Baseline CST**					
< 400 µm (n = 104)	96 (48%)	39 (40.6%)	57 (59.4%)	27 (58.7%)	19 (41.3%)
≥ 400 µm (n = 96)	104 (52%)	54 (51.9%)	50 (48.1%) p = 0.10	42 (64.6%)	23 (35.4%) p = 0.53
**Baseline DR severity**					
PDR (n = 87)	87 (43.5%)	48 (55.2%)	39 (44.8%)	35 (64.8%)	19 (35.2%)
NPDR (n = 113)	113 (56.5%)	45 (39.8%)	68 (60.2%) **p = 0.03**	34 (59.7%)	23 (40.4%) P = 0.58
**Foveal cysts**					
Present (n = 183)	183 (91.5%)	83 (45.4%)	100 (54.6%)	63 (61.2%)	40 (38.8%)
Absent (n = 17)	17 (8.5%)	10 (58.8%)	7 (41.2%) p = 0.29	6 (75.0%)	2 (25.0%) P = 0.36*
**NSD**					
Present (n = 53)	53 (26.6%)	27 (50.9%)	26 (49.1%)	16 (51.6%)	15 (48.4%)
Absent (n = 146)	146 (73.4%)	66 (45.2%)	80 (54.8%) p = 0.47	53 (66.3%)	27 (33.8%) p = 0.15
**DRIL**					
Present (n = 161)	161 (83.4%)	77 (47.8%)	84 (52.2%)	56 (59.6%)	38 (40.4%)
Absent (n = 32)	32 (16.6%)	12 (37.5%)	20 (62.5%) p = 0.29	9 (81.8%)	2 (18.2%) p = 0.13*
**ELM**					
Intact (n = 93)	93 (48.4%)	41 (44.1%)	52 (55.9%)	32 (74.4%)	11 (25.6%)
Disrupted or absent (n = 99)	99 (51.6%)	48 (48.5%)	51 (51.5%) p = 0.54	33 (54.1%)	28 (45.9%) **p = 0.04**
**EZ**					
Intact (n = 57)	57 (29.2%)	20 (35.1%)	37 (64.9%)	16 (69.6%)	7 (30.4%)
Disrupted or absent (n = 138)	138 (70.8%)	70 (50.7%)	68 (49.3%) p = 0.05	50 (59.5%)	34 (40.5%) p = 0.38
**PVD**					
Present (n = 11)	11 (5.5%)	5 (45.5%)	6 (54.6%)	5 (62.5%)	3 (37.5%)
Absent (n = 189)	189 (94.5%)	88 (46.6%)	101 (53.4%) p = 0.94	64 (62.1%)	39 (37.9%) p = 0.65*
**VMA/VMS**					
Present (n = 63)	63 (31.5%)	29 (46.0%)	34 (54.0%)	22 (62.2%)	9 (29.0%)
Absent (n = 137)	137 (68.5%)	64 (46.7%)	73 (53.3%) p = 0.93	47 (58.8%)	33 (41.3%) p = 0.23
**ERM**					
Present (n = 31)	31 (15.5%)	17 (54.8%)	14 (45.2%)	15 (68.2%)	7 (31.8%)
Absent (n = 169)	169 (84.5%)	76 (45.0%)	93 (55.0%) p = 0.31	54 (60.7%)	35 (39.3%) p = 0.52
**HRF**					
Present (n = 81)	81 (40.5%)	35 (43.2%)	46 (56.8%)	26 (60.5%)	17 (39.5%)
Absent (n = 119)	119 (49.5%)	58 (48.7%)	61 (51.3%) p = 0.44	43 (63.2%)	25 (36.8%) p = 0.77

*Fisher’s exact p-value.

*CST* central subfield thickness, *PDR* proliferative diabetic retinopathy, *NPDR* non-proliferative diabetic retinopathy, *NSD* neurosensory detachment, *DRIL* disorganisation of retinal inner layers, *ELM* external limiting membrane, *EZ* ellipsoid zone, *PVD* posterior vitreous detachment, *VMA* vitreomacular abnormalities, *VMS* vitreomacular separation, *ERM* epiretinal membrane, *HRF* hyperreflective foci.

## Discussion

This study evaluated early response of DMO to intravitreal aflibercept to decipher the baseline features of limited early responders so that newer drugs can be evaluated on a more appropriate patient cohort. We have to consider a few parameters in the trial design.

Firstly, any investigational product has to be evaluated against the most effective standard of care. Based on Protocol T, aflibercept is the appropriate comparator and we characterised the early limited responders with aflibercept therapy^4^.

The second consideration is the length of duration of a Phase 1 or proof of concept trial. Ideally, it should be kept as short as possible to tease out the characteristics of patients with limited response to aflibercept in the early phase. We chose the response after the loading phase because nearly all patients in real-life will receive the loading phase and therefore the response post-loading is not influenced by non-attendance or clinician decisions.

Thirdly, we defined the group of patients that has the most potential to improve VA with another agent. In this study, we found that 53.5% of eyes improved by less than 5 letters. This is higher than 44% of eyes in the aflibercept arm in Protocol T^8^, in keeping with the lower visual outcomes with anti-VEGF in real-life cohorts compared to clinical trials. Moreover, 40% of our study cohort presented with VA of 70 letters or better.

Based on different strata of baseline VA, our study clearly shows that a ceiling effect occurs at baseline VA 70 letters or above. As a small sample size is preferred in early phase studies, patients with baseline VA of 70 or more letters should ideally be excluded, as they are unlikely to improve significantly, whatever be the drug used. In addition, the ideal baseline VA group for trials is 35 to 54 letters where most variations in improvement of VA were observed. Furthermore, the 35–54-letter group had a higher proportion with limited functional and anatomical response than the 55–69 letter group. The 35–54 letter group encompassed almost a fifth of our study population.

In order to ensure that this inclusion–exclusion criterion is recruitment-friendly, we suggest that the baseline VA criterion of patients in trials on novel agents be increased to 35–69 letters. This is in line with Protocol T that showed clear superiority of aflibercept compared to bevacizumab or ranibizumab from the loading phase in the group with baseline VA less than 69 letters^4^. However, although in our study, both baseline VA categories 35–54 and 55–69 letters had similar mean VA gain of 7.6 letters after loading dose, more than half of those in the 55–69 letter group achieved the desirable outcome after the loading dose, compared to only 14% for the 35–54 letter group. So, if VA of 35–69 ETDRS letters is required for the inclusion criteria, it is prudent to further stratify by baseline VA into 35–54 and 55–69 letters.

We also defined early limited responders as those who did not achieve at least a 5-letter gain or CST reduction of < 10% after the loading phase. These criteria were chosen as there is only a modest correlation between visual acuity and CST^11^. Although most trials now enrol patients with Spectralis equivalent of 320 μm, our data showed that DMO patients with baseline CST < 400 μm do not respond functionally or anatomically as well as eyes with CST ≥ 400 μm at baseline. This was shown in RESTORE study subgroups^5^. In the United Kingdom, patients with CST < 400 µm are not treated despite visual impairment based on this RESTORE subgroup analysis and yet approximately 50% have persistent macular fluid at the end of 12 months of anti-VEGF therapy. Also, patients in the category of baseline 35–54 letters had the most numbers of limited responders in terms of CST reduction of < 10%. Therefore, an inclusion criterion for CST ≥ 400 μm is recommended in trials investigating novel agents for DMO. This conclusion is based on the point that with anti-VEGF agents, steroids and even laser treatment, we rely on CST as a response to treatment. It may be that novel agents may provide superior VA outcomes independent of the effect of CST and therefore, inclusion criteria including a CST cut off may not be required. This point should be considered when considering novel investigational products. Furthermore, although we found no correlation between change in VA and change in CST for the whole group, in the baseline VA 35–54 letter category, only 37.2% has complete resolution of fluid and 35% showed a reduction of less than 10%, showing that the two inclusion criteria of baseline VA 35–54 letters and CST of ≥ 400 μm are likely to capture the group of patients most likely to respond to another novel intervention, as this group had the most numbers of limited aflibercept responders.

Previous studies have found that eyes with subretinal fluid contributing to CST were more responsive to anti-VEGF therapy as compared to eyes without subretinal fluid^14^. Our study did not show any differential response in eyes with neurosensory detachment.

In terms of anatomical changes seen on OCT, our study found that those with foveal cysts and those with disruption or absence of ellipsoid zone layer (EZ) were less likely to have CST reduction of 10% or more. Although these anatomical changes can be used as imaging biomarkers to predict response, EZ layer is often masked by shadowing of intraretinal fluid at initiation of treatment and may re-constitute or re-appear post macular fluid resolution. Loss of integrity of EZ layer is a poor prognostic marker of VA but it is difficult to ascertain the integrity of EZ layer in the presence of cystoid macular oedema adjacent to the outer retina and is therefore best to avoid this point within the inclusion–exclusion criteria.

When we consider the diabetic retinopathy status, a higher proportion of patients with co-existing PDR did not achieve 5 letter gain after 3 aflibercept loading doses but this group did not show any difference in response in reducing CST by more than 10% suggesting that this difference is not related to presence of DMO. This effect may be due to co-existent macular ischaemia or abnormalities at vitreo-retinal interfaces such as epiretinal membrane, which is more prevalent in eyes with PDR than NPDR. Therefore, in future trials, a sub-analysis of outcomes based on DR status is recommended.

The strength of this study is that we have assessed the response of 3 loading injections of aflibercept in patients in clinical practice and therefore our findings are more generalisable compared to clinical trial cohorts. Our findings on CST and VA correlation resemble that of previous similar analysis done in clinical trials further justifying that the observations noted in this study may be used to plan future studies.

This study is limited in that it did not assess potential associations of DMO with other factors such as the presence of macular ischemia and level of glycaemic control, which have both been shown to be directly associated with poor VA outcomes^14^.

In conclusion, we have characterised the baseline features of limited early responders to aflibercept loading dose to allow newer drugs to be evaluated on a more appropriate patient cohort. We found that those with baseline VA between 35–54 ETDRS letters and CST >  = 400 μm are the ideal cohort that will most likely benefit from trials with other agents.
